# The oncological and obstetric results of radical trachelectomy as a fertility-sparing therapy in early-stage cervical cancer patients

**DOI:** 10.1186/s12905-022-01990-w

**Published:** 2022-10-26

**Authors:** Tao Chen, Jia Li, Yan Zhu, An-Wei Lu, Li Zhou, Jian-San Wang, Ying Zhang, Jun-Tao Wang

**Affiliations:** 1 Department of Gynecology, Maternal and Child Health Care Hospital of Guiyang, NO.63 Ruijin South Road, 561000 Guiyang, Guizhou China; 2 Department of Gynecology, Shenzhen Hospital Affiliated to Southern Medical University, NO.1333 Xinhu Road, 518000 Shenzhen, China; 3 Department of Reproductive Center, Maternal and Child Health Care Hospital of Guiyang, NO.63 Ruijin South Road, 561000 Guiyang, Guizhou China

**Keywords:** Cervical cancer, Fertility preservation, Radical trachelectomy, Neoadjuvant chemotherapy

## Abstract

**Purpose:**

This study explored the oncological and obstetric results of radical trachelectomy (RT) in early-stage cervical cancer patients.

**Methods:**

A retrospective analysis was conducted the oncological and obstetric results of 23 patients with early cervical cancer (stages IA2–IB3; International Federation of Gynecology and Obstetrics, 2018) who underwent RT in The Maternal and Child Health Care Hospital of Guiyang, China, from October 2004 to September 2018.

**Results:**

23 patients had cervical tumors of the squamous cell carcinoma histological type. All 23 patients retained reproductive function. The mean follow-up time was 112.87 ± 55.75 (36–199) months. The median tumor size was 2.00 ± 1.35 cm (imperceptible to the eyes 5.00 cm). No recurrence was observed in any of the patient cases. Among the patients with a tumor size > 4 cm (up to 5 cm), three patients who wished to preserve fertility accepted RT following neoadjuvant chemotherapy The pregnancy outcomes were as follows: 8 cases (47.06%) out of 17 cases who attempting pregnancy conceived 12 times.First-trimester abortion and the voluntary abandonment of pregnancy occurred in 4 cases (33.33%), respectively, one patient performed deliberate termination at 24 weeks of gestation. Second-trimester abortion occurred in three cases (25.0%) for chorioamnionitis. Premature delivery at 32 weeks occurred in one case (8.33%).

**Conclusion:**

Radical trachelectomy is a safe and effective treatment for women with early-stage cervical cancer preserving fertility biology. Patients with a cervical tumor sized > 4 cm can be pregnant after neoadjuvant chemotherapy and RT. Accordingly, this treatment is worthy of further exploration.

## Introduction

Cervical cancer is the fourth most common malignant diseases among women worldwide [1]. Its incidence and mortality rates is related to the economic level of a region,because the incidence rate and mortality of cervical cancer are more concentrated in regions with medium / low human development index [2].

China has a large cervical cancer patient population as a developing country with a large population. A trend of early onset of this disease in the younger population was found, with the continued development and popularization of cervical cancer screening technology [3]. Epidemiology surveys indicate that the average age of cervical cancer onset has decreased by 5–10 years compared with pre-2000.An increasing number of patients are being diagnosed with the disease during their reproductive period [3, 4]. At the time of diagnosis, a growing number of patients had had only one child or had never been pregnant [4]. In these cases, the preservation of reproductive function is likely to have a positive long-term impact on the lives and psychological well-being of patients.

Radical hysterectomy (RH) is currently the standard procedure for the treatment of cervical cancer. Despiting its high cure rate, the treatment also eliminates patients’ fertility function due to the removal of the uterus. Therefore, a safe and effective procedure opting to preserve the reproductive function of patients will be a breakthrough in the treatment of cervical cancer.

Regarding the development characteristics of cervical cancer, parauterine and vagina types are more typically observed, while uterine and uterine adnexa cancer types are rare [5]. These characteristics provide a feasible theoretical basis for conservative surgery for cervical cancer to preserve reproductive function. Burghardt [6] presented a fertility-preserving surgical approach for early-stage cervical cancer in 1977 but this was never implemented.

In 1987, Dargent et al. [7] implemented laparoscopic pelvic lymphadenectomy (LPL) combined with vaginal radical trachelectomy (VRT), which subsequently led to a successful full-term pregnancy, which was the first success in preserving fertility function in patients with early-stage cervical cancer. Following researches conducted by Smith [8], Shepherd [9], and others presented successful techniques for performing RT via different pathways, such as abdominal, laparoscopic, and robot-assisted procedures. These types of surgeries can completely remove cervical cancer lesions and preserve the patient’s uterus and the uterine adnexa.

Significant progress has been made in the treatment of early-stage cervical cancer. However, since RT surgery is not the standard treatment for cervical cancer and requires strict admittance criteria, it has not been conducted for a wide range of patients. In addition, this type of surgery was implemented in China later than in developed countries; thus, the proportion of these patients is even smaller in China. Therefore, study about the experiences and outcomes of this surgical procedure is particularly significant in our country.

The researchers’ institution is a tertiary hospital in China where the RT procedure was first conducted in 2004. Since then, the procedure has been successfully performed for 23 patients who wished to preserve their fertility. Three of these patients received neoadjuvant chemotherapy (NACT) for cervical tumors sized > 4 cm, and RT was performed after the re-evaluation of chemotherapy results. After an extended follow-up period, we collected and retrospectively analyzed the relevant information of these patients to assess the oncological and obstetric outcomes after RT in early-stage cervical cancer. Additionally, the authors also explored the oncological safety and clinical outcomes of RT following NACT in female patients with cervical tumors sized > 4 cm.

## Methods

### Patients

Clinical and follow-up data were collected from 23 patients who received RT to preserve fertility function in the Maternal and Child Health Care Hospital of Guiyang from October 2004 to September 2018. All the patients had been diagnosed with cervical cancer by cervical biopsy via colposcopy or cervical conical resection. A pelvic examination was performed by two chief or associate chief gynecological oncologists to assess the tumor size, and pelvic magnetic resonance imaging (MRI) was conducted to assess the size of the lesions and exclude suspected parauterine infiltration and swollen lymph nodes in the pelvic area. Additionally, preoperative communication was performed with all the patients to ensure that they had a strong desire to preserve reproductive function. These patients were informed that the procedure was not a standard technique for cervical invasive cancer; the patients were also aware of the related risks involved to ensure that they were fully informed before consenting to undergo the procedure. Finally, as defined by the International Federation of Gynecology and Obstetrics (FIGO) stages (see Table 1), 23 patients were included, comprising 2 cases of stage IA2, 16 cases of IB1, 2 cases of IB2, and 3 cases of IB3.

**Table 1:** Surgical staging of cervical cancer (2018)

Stages	Standard
I	The carcinoma is strictly confined to the cervix (extension to the uterine corpus should be disregarded)
I A	Invasive carcinoma that can be diagnosed only by microscopy, with maximum depth of invasion < 5 mm
I A1	Measured stromal invasion < 3 mm in depth
I A2	Measured stromal invasion ≥ 3 mm and < 5 mm in depth
I B	Invasive carcinoma with measured deepest invasion ≥ 5 mm (greater than Stage IA), lesion limited to the cervix uteri
I B1	Invasive carcinoma ≥ 5 mm depth of stromal invasion, and < 2 cm in greatest dimension
I B2	Invasive carcinoma ≥ 2 cm and < 4 cm in greatest dimension
I B3	Invasive carcinoma ≥ 4 cm in greatest dimension
II	The carcinoma invades beyond the uterus, but has not extended onto the lower third of the vagina or to the pelvic wall
II A	Involvement limited to the upper two-thirds of the vagina without parametrial involvement II a1 Invasive carcinoma < 4 cm in greatest dimension II a2 Invasive carcinoma ≥ 4 cm in greatest dimension
II B	With parametrial involvement but not up to the pelvic wall
III	The carcinoma involves the lower third of the vagina and/or extends to the pelvic wall and/or causes hydronephrosis or nonfunctioning kidney and/or involves pelvic and/or para-aortic lymph node
III A	The carcinoma involves the lower third of the vagina, with no extension to the pelvic wall
III B	Extension to the pelvic wall and/or hydronephrosis or nonfunctioning kidney (unless known to be due to another cause)
III C	Involvement of pelvic and/or para-aortic lymph nodes, irrespective of tumor size and extent (with r and p notations)
III C1	Pelvic lymph node metastasis only
III C2	Para-aortic lymph node metastasis
IV	The carcinoma has extended beyond the true pelvis or has involved (biopsy proven) the mucosa of the bladder or rectum. (A bullous edema, as such, does not permit a case to be allotted to Stage IV)
IV A	Spread to adjacent pelvic organs
IV B	Spread to distant organs

All the pathological types were cervical squamous cell carcinoma (SCC) with primary lesions located in the cervix vagina. The clinical characteristics of the patients are shown in Table 2.

**Table 2:** The clinical characteristic and operative details of 23 patients

	Average (range) or N (%)
Age (years)	32.22 ± 4.09 (26–40)
Follow-up (months)	112.87 ± 55.75 (36–199)
Fertility status	
With children	9 (39.13%)
Nulliparous	14 (60.87%)
FIGO stage	
IA2	2 (8.70%)
IB1	16 (69.57%)
IB2	2 (8.70%)
IB3	3 (13.04%)
Degree of differentiation	
Highly differentiation	8 (34.78%)
Moderately differentiation	11 (47.83%)
poorly differentiation	4 (17.39%)
The max diameter of tumor (cm)	2.00 ± 1.35 (invisible by eyes-5.00)
Operation methods	
EPL + VRT	3 (13.04%)
LPL + VRT	9 (39.13%)
LPL + LRT	11 (47.83%)
Operation duration (minutes)	203.91 ± 52.66 (150–420)
Intraoperative blooding (ml)	345.65 ± 376.26 (100–2000)
Iymph node count	21.52 ± 7.06 (10–33)
Postoperative complications	
Urinary retention	1 (4.35%)
Tubal vaginal fistula	1 (4.35%)
Intrauterine adhesions	1 (4.35%)

EPL: extraperitoneal pelvic lymphadenectomy

VRT: vaginal radical trachelectomy

LPL: laparoscopic pelvic lymphadenectomy

LRT: laparoscopic radical tracheletomy

### Preoperative treatment

Three patients with stage IB3 (tumor diameter, 5 cm (both), exophytic) who insisted on fertility preservation received platinum-based NACT preoperatively. Whether RT was appropriate based on the effect of chemotherapy.

### Surgical procedures

The procedure comprised pelvic lymph node dissection and RT. There are two methods of pelvic lymph node dissection,one is extraperitoneal (EPL) and another is LPL node resection. Common iliac, external iliac, deep inguinal, internal, and obturator lymph nodes were isolated and removed along the iliac vessels. Sample sections were frozen, and a pathological examination of the bilateral pelvic lymph nodes was conducted. Negative results of lymph nodes were followed by RT, while a positive result was followed by RH instead.

We performed RT using two different methods, i.e., VRT and laparoscopic RT (LRT). The separated vaginal wall was cut circularly 3 cm away from the outer opening of the isthmus, and the cervix was removed 1 cm below and away from the inner opening of the cervix. The removed cervix was subsequently sent to frozen section for examination. Radical trachelectomy can be performed uninterruptedly when the incision margin is 8 mm away from the lesion; if the distance between the cutting edge and the upper edge of the lesion is less than 5 mm, the remaining cervix should be removed by 3–5 mm; if the incision margin is involved, RH should be performed. Finally, the cervix was sutured using the Stumdorf method, and an intrauterine drainage tube was kept in place for a week to prevent cervical adhesion. Cerclage was a consideration according to the patient’s aspiration and the situation during the operation.

### Postoperative adjuvant therapy

According to the pathological results, the patients who showed risk factors for lymphatic vascular-space invasion, deep matrix invasion, and low differentiation degree were treated with platinum-based combination chemotherapy for 4–6 courses of adjuvant chemotherapy after surgery.

### Postoperative follow-up

Based on the cervical cancer follow-up criteria, the frequency of follow-up should be appropriately increased when necessary. The follow-up interval was 3 months within 2 years after the surgery, 6 months between 2 and 5 years after the procedure, and 1 year after 5 years following the operation. If abnormal conditions, such as atypical vaginal bleeding or menstrual changes were observed, the patient was immediately referred to a doctor. Routine follow-up included gynecological examination, Thinprep cytologic test (TCT), a human papillomavirus test, and gynecological ultrasonography. Pelvic and abdominal MRIs should also be conducted if necessary. The special content is sex life and pregnancy guidance.

### Statistical analysis

The collected data were analyzed using the SPSS Statistics 23.0 (Chicago, Illinois, USA) software program. The measurement data (age, surgery duration, lymph node count, anal intraoperative blooding, follow-up time, etc.) were measured as the mean ± standard deviation, and the data counts (intraoperative complication, postoperative complication, recurrence, pregnancy, etc.) were displayed as rates.

## Results

### Surgery and complications

Three patients with IB3-stage cervical cancer who insisted on preserving reproductive function received NACT before surgery. After two courses of chemotherapy, the tumor size of each patient who received neoadjuvant chemotherapy had shrunk more than 50%. The maximum tumor diameter in these 3 cases was reduced to 1.8, 1.5, and 2.0 cm, respectively. Finally, 23 patients (100%) underwent RT and reproductive function was successfully preserved. Six patients underwent cervical cerclage during surgery. The surgical methods, the average operation duration, the average intraoperative bleeding, and the average lymph node count are shown in Table 1.

During the operation, no intraoperative complications occurred in any of the patients, except for one patient had intraoperative bleeding (2,000 ml) who got severe pelvic and abdominal adhesion. This patient developed a complication in the form of a left tubal vaginal fistula after the surgery.She completely recovered 4 months after a double J tube implantation. In addition, one patient developed urinary retention after surgery. Following bladder function recovery exercise, the urinary tube was removed, and spontaneous urination resumed 21 days after surgery. One patient developed intrauterine adhesions 18 months after surgery and underwent hysteroscopic adhesion separation.

### Postoperative supplementary treatment

The pathological examination of intraoperative frozen sample sections and postoperative sample paraffin sections of the 23 patients showed no tumor metastases in the pelvic lymph nodes. No residual lesions were found in the cervical stump tissue, and no invasive lesions were found within 8 mm of the cervical cutting edge. Only one patient’s postoperative pathological examination results showed lymph node metastasis. The doctors recommended postoperative complementary therapy with concurrent chemoradiotherapy. However, the patient refused radiotherapy for personal reasons and only received two courses of supplementary chemotherapy after the surgery (the patient had received two courses of NACT before the procedure). In total, 8 patients (28.6%) with risk factors received platinum-based combination chemotherapy (4–6 courses)Table 3.

**Table 3:** the details of postoperative supplementary treatment of 8 patients with risk factors

Risk factors	Chemotherapy regimens
poorly differentiated SCC, scattered in the myometrium of the cervix	DC
poorly differentiated SCC, invading the middle myometrium of the cervix	DC
TD>4 cm	TC
TD>4 cm, poorly differentiated SCC, invading the middle myometrium of the cervix	TC
TD>2 cm, invading the middle myometrium of the cervix	DC
tumor thrombus can be seen in the lymphatic vessel	TC
TD>4 cm, poorly differentiated SCC, a lymph node metastasis	TP
TD>2 cm	DC

TD: tumor diameter

SCC: squamous cell carcinoma

DC: docetaxel + carboplatin

TC: taxol + carboplatin

TP: taxol + cis-platinum

^a^ the patient who accepted neoadjuvant chemotherapy

### Oncological and obstetric outcomes

None of the 23 patients were lost to follow-up. The average follow-up period was 112.87 ± 55.75 (36 ~ 199) months. No recurrence was observed in any of the patients (recurrence rate, 0%). All 23 patients were satisfied with their sexual life after the surgery (sexual life satisfaction, 100%). One patient underwent bilateral tubal ligation due to severe pelvic and abdominal adhesion and bilateral fallopian tube deformation during the operation. The presence of infertility factors was confirmed in six patients (26.09%) through pregnancy preparation examination; the remaining 16 patients (69.56%) did not undergo pregnancy preparation examination and, as such, the presence/absence of infertility factors among them remained unknown.

Six patients (26.09%) halted their attempts to become pregnant for reasons of divorce, fear of tumor recurrence, ovarian hypofunction, or because they had given birth before cancer. A total of 17 patients (73.91%) attempted to become pregnant. 8 of them got preganat12 times (pregnancy rate, 47.06%), 10 of which were spontaneous pregnancies, and 2 were the result of assisted reproduction. Two cases (16.67%) experienced spontaneous abortion for unknown reasons and 2 (16.67%) cases had a missed abortion at 8 weeks gestation. Three (25%) cases experienced spontaneous abortion at 16, 17, and 19 weeks gestation, respectively, and the cause of abortion was chorioamnionitis. One case (8.33%) had a spontaneous mid-term induction of labor to terminate their pregnancy at 6 months of gestation. One case (8.33%) had a vaginal delivery at 32 weeks gestation, and the cause of premature delivery was cervical insufficiency. The child is healthy until this time. Ten people (58.82%) gave up conceiving, for one hand, people who had failed pregnancy attempts and refused assisted reproductive consultation due to family concept, economic factors, fear of tumor recurrence or had given birth before cancer. At present, 7 patients are still actively trying to become pregnant, and 3 patients are receiving assisted reproductive counseling. The fertility and obstetric outcomes are shown in Table 4.

**Table 4:** Fertility and obstetric outcomes following 23 patients

	N(%)
Sexual satisfaction	23(100%)
Had pregnancy attempts	17(73.91%)
Still have pregnancy attempts	7(30.43%)
Got pregnancy	8(47.06%)
Pregnancy ways	
Spontaneous	10(83.33%)
In-vitro fertilization	2(16.67%)
Infertility factors	
Ovarian	2(8.70%)
fallopian tubes	5(21.74%)
Unknown	16(69.56%)
obstetric outcomes	
First-trimester loss	4(33.33%)
Second-trimester loss	3(25.0%)
Premature delivery(live birth)	1(8.33%)
Voluntary abortion	4(33.33%)

## Discussion

In light of the advanced techniques to detect early-stage disease, more gynecological cancers are being detected at an early stage. It is crucial to preserve reproductive potential of women affected by gynecological cancers in their reproductive years. Many scholars have confirmed through a large number of clinical trials that individualized programs can be implemented for women with early gynecological cancer who have strong reproductive function, such as endometrial cancer [10] and cervical cancer [11].

Only a small degree of parauterine involvement during the early stage of cervical cancer and a low risk of recurrence according to the current literature. A review conducted by Schmeler [12] including research ranging from 1970 to 2010 suggested that parauterine infiltration was reflected in only 1% (low risk) of early cervical cancer patients (IA2, IB1, tumor size < 2 cm). Furthermore, the lymph node and metastases rates of parauterine vessels in early cervical cancer were also low. The National Comprehensive Cancer Network (2020) guidelines on cervical cancer propose the indications for RT surgery as follows [5]: patients have strong fertility requirements following adequate consultation; fertility-sparing therapy is suitable for patients with cervical SCC in stages IA2–IB1 (tumor size < 2 cm) in principle, but common adenocarcinoma is not an absolute contraindication; fertility preservation is not recommended for patients with high- or moderate-risk factors.

Conversely, studies have also shown that RT can remove the same parametrial tissue as RH. Han et al. [13] systematically analyzed clinical randomized controlled trials of RT and RH from 1994 to 2010, and the results indicated no difference between RT and RH in 5-year overall survival, 5-year disease-free survival, or intraoperative and postoperative complications. Compared with RH, RT has more advantages concerning intraoperative blood loss, blood transfusion rate, and postoperative hospital stay. Xu et al. [14] conducted a meta-analysis of 587 patients with early cervical cancer who were treated with RT or RH. The results showed no significant discrepancy in recurrence rate, a 5-year progression-free survival rate, and a 5-year overall survival rate between the two groups. In the present study, 20 of the 23 patients fully complied with the requirements of the above indications, and no recurrence was observed. The mean follow-up was 112.87 months; 15 patients were followed up for longer than 5 years. These results demonstrated that it was safe for patients who meet the inclusion criteria to receive RT to preserve reproductive function.

The indications for RT are not consistent, however, and whether the treatment is feasible for patients with a tumor diameter that is > 2 cm remains controversial. Rutledge et al. [14] conducted a statistical analysis of patients with stage IB cervical cancer to show that lymphatic vascular-space invasion and deep stromal invasion, rather than tumor size, had prognostic significance in multivariate analysis. Hence, RT appears to be a reasonable treatment approach if there is no interstitial infiltration in patients with exophytic tumor lesions, even for the IB2 stage with a tumor diameter that is > 2 cm. In a study conducted by Li. et al. [15], 35 patients with cervical cancer at stage IB1 (tumor diameter was > 2 cm; FIGO 2009) underwent an abdominal RT (ART) to preserve fertility and did not receive adjuvant treatment after surgery. The average follow-up period was 30.2 months without recurrence.

A large number of studies ^[11, 18, 19]^ have shown that for patients with a large tumor volume, NACT can be given locally to effectively reduce the tumor volume for complete resection of a tumor subsequently;and the tumor-free area at the edge of the incision can be increased. Marchiole et al. [18] used NACT combined with LPL and VRT to preserve fertility in 7 patients with stage IB–IIA1 cervical cancer (tumor size 3.0–4.5 cm; FIGO, 2009). The tumor completely subsided in 4 patients and was reduced by 50% or more in 3 patients following NACT. Finally, 6 patients successfully retained fertility with a median follow-up of 22 months and there was no recurrence. One patient tried to become pregnant after therapy and was successful. Moreover, Tesfai et al. [19] conducted a retrospective analysis, including 19 patients who had been treated with NACT for a cervical tumor that was > 2 cm (range, 3.5–6.0 cm) before ART; 15 patients’ fertility was successfully preserved. The authors concluded that NACT followed by fertility-sparing surgery may be a viable and safe option for selected patients with cervical tumors that were > 2 cm.

In the current study, three patients with stage IB3 (a tumor that was sized > 4 cm; FIGO, 2018) were included, based on their strong desire to preserve their fertility. They accepted NACT to reduce the tumor diameter, thereby establishing the conditions for fertility-sparing surgery following sufficient evaluation. After two cycles of NACT, the tumor volume had decreased by more than 50%. The patients then underwent fertility-sparing surgery. They also received 2–4 courses of postsurgical chemotherapy. To date, 2 patients have had disease-free survival for more than 5 years, and one patient has remained free of recurrence for more than 4 years.It appears feasible for patients with stage IB3 cervical cancer, who show effective results following NACT, as an approach for preserving fertility. However, since the research on fertility preservation for patients with a large tumor diameter is still in the exploratory stage, this must be explored via large-scale clinical trials furthurly.

The most common complications during RT were vascular injury and surrounding organ injury, e.g., to the bladder, ureter, and intestine. No intraoperative complications occurred in our study, except for one patient who experienced severe pelvic and abdominal adhesion, which led to a significant amount of bleeding (2,000 ml). Two patients experienced postoperative complications, including bladder dysfunction and intrauterine adhesions. Under the guidance of bladder function exercise, patient with postoperative bladder dysfunction resumed spontaneous micturition 21 days after the surgery. We performed hysteroscopy and adhesion-separation treatment after excluding cervical stenosis on the patient who has intrauterine adhesion. Following the surgery, the stomach cramps disappeared, and menstruation returned to normal.

The preservation of fertility after surgery is as important as the survival rate for patients who have undergone RT. Currently, reports present variable results on obstetric outcomes after fertility-preserving therapy for early cervical cancer. Reports compiled by Dargent [7], Plante [18], and Shepherd [19] showed that the pregnancy rate was higher than 50%, while the live birth rate was 52%, 72%, and 50%, respectively. The results of these studies showed that RT preserved high pregnancy and live birth rates. In China, Li et al. [20] conducted research that included 360 patients who underwent VRT; 149 patients attempted to become pregnant following treatment and a subsequent pregnancy rate of 17.4% was achieved and a live birth rate of 63.3%. Compared with international data, the pregnancy rate was lower, and the live birth rate was similar.These results discrepancies may be related to case selection, surgical technique, follow-up time, sample quantity, and the degree level of assisted reproductive technology. Data compiled in our study showed a similar pregnancy rate (47.06%) and a lower live birth rate (8.33%), which may be associated with the small sample size, patients rejecting assisted reproductive technology treatment, and statistical deviation caused by the spontaneous abandonment of pregnancy. one patient selected to induce abortion of a mid-trimester pregnancy.

The risk of pregnancy-related complications, such as the preterm or premature rupture of membranes, miscarriage, and preterm delivery, were increased due to altered cervical tissue morphology and the absence of protective mechanisms after RT [21]. The miscarriage rate at the middle stage of pregnancy (8.6%) was twice as the rate observed among the general population.Most of them occurred due to the premature rupture of membranes caused by infection [22]. In the present study, three cases of second-trimester miscarriage were caused by chorioamnionitis. The high rate of preterm delivery among pregnant patients was another important concerns, the reason for which is believed to be mechanical or ascending infection, or both [23]. Some researchers believed that it is unnecessary to excise all of the cervical tissue during surgery and that it is beneficial to retain cervical tissue of up to 5–10 mm to reduce the incidence of premature birth as much as possible [24]. Some researchers put forward that cerclage could be adopted during surgery to prevent cervical insufficiency [20, 25]. However, studies have also posited different opinions about the timing of cerclage, claiming it may cause cervical stenosis and become a factor related to infertility. According to present research results, cervical ligation during the surgery is not recommended [22, 26]. In our study, two patients who underwent intraoperative prophylactic cerclage suffered a miscarriage. One patient who underwent cerclage at 14 weeks of gestation and delivered a live baby in parturition at 32 weeks vaginally. This may suggest that prophylactic cervical cerclage during RT does not reduce the risk of a postoperative miscarriage occurring.

RT surgery is currently divided into VRT, ART, LRT, and robot-assisted laparoscopic RT (RRT). Research data indicate that the pregnancy outcomes obtained by different routes of RT are different. [27–30]. A large number of clinical studies have shown that Laparoscopic pelvic lymphadenectomy (LPL) is safe and feasible for patients with cervical cancer. LPL is safe and feasible even for women with early cervical cancer during pregnancy to evaluate the disease and make the next diagnosis and treatment plan, and can obtain better tumor and pregnancy outcomes ^[31, 32]^.In our study, the postoperative pregnancy rate was higher in patients who underwent EPL combined with VRT (2/3, 66.67%) compared with patients who underwent LPL combined with VRT/LRT (6/20, 30.0%); this may have been related to fewer pelvic adhesions resulting from the EPL combined with VRT surgery method.

The data analyzed from the current study may lack statistical significance due to its small sample size. Regardless of the surgical method ,its careful execution and the minimal trauma will be conducive to optimize the postoperative pregnancy rate. It is worth emphasizing that RT, as a cancer treatment method, must ensure the safety of oncological results as a premise, rather than pursuit hihger postoperative pregnancy rate blindly.

## Conclusion

Radical trachelectomy is feasible as a fertility preservation treatment for young patients who are diagnosed with early cervical cancer and can effectively preserve fertility function. In addition, NACT combined with RT offers the possibility of preserving fertility function for patients with cervical cancer when a tumor is sized > 4 cm. However, related research is still in the exploratory stage, the results of the present study must be explored via large-scale clinical trials, which are worthy of further exploration.

## Data Availability

The datasets used and/or analysed during the current study available from the corresponding author on reasonable request. We declared that materials described in the manuscript, including all relevant raw data, will be freely available to any scientist wishing to use them for non-commercial purposes, without breaching participant confidentiality.

## Declarations

This manuscript conformed the Enhancing the QUAlity and Transparency Of health Research (EQUATOR) network guidelines.

## Abbreviations

FIGO: International Federation of Gynecology and Obstetrics
SCC: squamous cell carcinoma
RH: radical hysterectomy
LPL: laparoscopic pelvic lymph node resection
VRT: vaginal radical trachelectomy
RT: radical trachelectomy
NACT: neoadjuvant chemotherapy
MRI: magnetic resonance imaging
EPL: extraperitoneal pelvic lymph node resection
LRT: laparoscopy radical trachelectomy
NCCN: National Comprehensive Cancer Network
LVSI: lymphatic vascular space invasion
DSI: deep stromal invasion
ART: abdominal radical hysterectomy
PROM: preterm premature rupture of membranes
RRT: robot-assisted laparoscopic radical tracheletomy
